# A Machine Learning Challenge: Detection of Cardiac Amyloidosis Based on Bi-Atrial and Right Ventricular Strain and Cardiac Function

**DOI:** 10.3390/diagnostics12112693

**Published:** 2022-11-04

**Authors:** Jan Eckstein, Negin Moghadasi, Hermann Körperich, Elena Weise Valdés, Vanessa Sciacca, Lech Paluszkiewicz, Wolfgang Burchert, Misagh Piran

**Affiliations:** 1 Institute for Radiology, Nuclear Medicine and Molecular Imaging, Heart and Diabetes Center North-Rhine Westphalia, Ruhr-University of Bochum, 32545 Bad Oeynhausen, Germany; 2 Department of Engineering Systems & Environment, University of Virginia, Charlottesville, VA 22904, USA; 3 Clinic for Electrophysiology, Heart and Diabetes Center North-Rhine Westphalia, Ruhr-University of Bochum, 32545 Bad Oeynhausen, Germany; 4 Clinic for Thoracic and Cardiovascular Surgery, Heart and Diabetes Center North-Rhine Westphalia, Ruhr-University of Bochum, 32545 Bad Oeynhausen, Germany

**Keywords:** artificial intelligence, cardiovascular magnetic resonance, cardiac strain, support vector machine, cardiac amyloidosis

## Abstract

Background: This study challenges state-of-the-art cardiac amyloidosis (CA) diagnostics by feeding multi-chamber strain and cardiac function into supervised machine (SVM) learning algorithms. Methods: Forty-three CA (32 males; 79 years (IQR 71; 85)), 20 patients with hypertrophic cardiomyopathy (HCM, 10 males; 63.9 years (±7.4)) and 44 healthy controls (CTRL, 23 males; 56.3 years (IQR 52.5; 62.9)) received cardiovascular magnetic resonance imaging. Left atrial, right atrial and right ventricular strain parameters and cardiac function generated a 41-feature matrix for decision tree (DT), k-nearest neighbor (KNN), SVM linear and SVM radial basis function (RBF) kernel algorithm processing. A 10-feature principal component analysis (PCA) was conducted using SVM linear and RBF. Results: Forty-one features resulted in diagnostic accuracies of 87.9% (AUC = 0.960) for SVM linear, 90.9% (0.996; Precision = 94%; Sensitivity = 100%; F1-Score = 97%) using RBF kernel, 84.9% (0.970) for KNN, and 78.8% (0.787) for DT. The 10-feature PCA achieved 78.9% (0.962) via linear SVM and 81.8% (0.996) via RBF SVM. Explained variance presented bi-atrial longitudinal strain and left and right atrial ejection fraction as valuable CA predictors. Conclusion: SVM RBF kernel achieved competitive diagnostic accuracies under supervised conditions. Machine learning of multi-chamber cardiac strain and function may offer novel perspectives for non-contrast clinical decision-support systems in CA diagnostics.

## 1. Background

Cardiac amyloidosis (CA) is a rare restrictive cardiomyopathy, characterized by an uncontrolled amyloid protein deposition that exacerbates organic function [1]. Early CA diagnostics is usually hindered by indistinguishable clinical presentation and greater awareness of more prevalent hypertrophic diseases and heart failure syndromes. Thus, CA can be considered underdiagnosed, consequently delaying necessary therapeutic measures, reducing quality of life and worsening clinical prognosis [2,3].

Cardiac magnetic resonance (CMR) imaging plays an important role in CA diagnosis and CA differentiation from other hypertrophic diseases such as hypertrophic cardiomyopathy (HCM). Despite clinical and radiological advances, CA remains a challenging diagnosis usually confirmed by specialized centers with significant delay in the patient’s clinical odyssey. The state-of-the-art CA diagnosis via CMR is ideally substantiated by diffuse sub-endocardial or transmural late gadolinium enhancement (LGE), elevated myocardial native T1, elevated extracellular volume fraction and the inability of myocardial signal suppression in phase-sensitive inversion recovery (PSIR) LGE sequences [4]. Their individual incremental diagnostic and prognostic value has been profoundly investigated [5,6,7,8,9,10]. Therefore, the left ventricle serves as the primary chamber for diagnostic validation.

However, recent investigations have shown that cardiac strain provides discriminative value for all cardiac chambers and thus may support differentiation of CA from hypertrophic cardiomyopathy (HCM) and healthy control subjects (CTRL) [6,11,12,13,14,15]. Based on previous observations [11,12,14,15,16] and in the hope of optimizing CMR diagnostic accuracy for CA detection, we hypothesize that strain measurements of the diagnostically less popular cardiac chambers along with general cardiac functional parameters may achieve a high CA diagnostic accuracy. Cardiac strain parameters would thus solely derive from both atria and the right ventricle. This study was conducted primarily as a scientific challenge, elucidating the diagnostic value of multi-chamber wall deformation.

The complexity of this diagnostic approach, encompassing a wide spectrum of parameters, demands the computational gold standard termed machine learning. Machine learning is a function of artificial intelligence, used as a promising alternative to overcoming the limitations of traditional prediction approaches [17]. Recent supervised and unsupervised machine learning approaches have proven highly valuable for the classification, regression and prediction of complex datasets in all industrial sectors, including the medical field of CA diagnostics [3,18,19]. For example, promising machine learning aspects have been based on CA-specific laboratory profiles of heart failure patients [19] or CA-associated ECG mapping [18]. This study utilized a support vector machine (SVM) algorithm to investigate the diagnostic performance of CMR cardiac strain and functional parameters from the left atrium (LA), right atrium (RA) and right ventricle (RV) for biopsy-validated CA patients, contrasting them to corresponding data from HCM patients and CTRL subjects. Utilizing 41 strain and functional parameters, we assess the diagnostic performance of SVM for CA detection. For the purpose of efficient and cost-effective clinical practice, an additional machine learning algorithm was conceptualized using the 10 most valuable diagnostic parameters available after the post-processing of a standard CMR examination.

## 2. Methods

### 2.1. Study Population

This study has a retrospective single-center observational design. The inclusion criteria called for biopsy-validated CA patients and their subtypes. CA patients had received a routine CMR according to standard diagnostic protocol with adequate imaging quality. HCM patients were enrolled upon confirmed diagnosis in accordance with ESC guidelines 2014 [20] and AHA guidelines 2020 [21]. A group of healthy volunteers aged over 50 years was included for improved comparison. Written informed consent was obtained from all participants prior to the examination. Exclusion criteria for CTRL subjects included a clinical history of cardiovascular disease and surgery, medication for cardiovascular or metabolic disorders, associated risk factors, and contraindications for CMR. If CMR imaging demonstrated myocardial abnormalities, aortic ectasia, pulmonary trunk dilation, valvular heart disease, ischemic heart disease or signs of cardiomyopathy the control individuals would be excluded. All examinations were done in accordance with the 1964 declaration of Helsinki and the study was approved by the local ethics committee (Ethik-Kommission der Medizinischen Fakultät der Ruhr-Universität Bochum; registration number 2017-238).

### 2.2. Variable Selection

In adherence to the study objective, the included variables were selected based on a profound array of strain and strain rates of the RA, LA and RV. For both atria, this resulted in reservoir, conduit and booster longitudinal strain and strain rate in the two- and four-chamber view. For the right ventricle, circumferential, radial and longitudinal strain and strain rate were quantified in the short-axis and four-chamber view. Lastly, in addition to demographic parameters such as age and body surface area, general cardiac functions were included, such as age, body surface area, heart rate, right heart volumetrics, and left and right ventricular ejection fraction were included. In sum, 41 different variables were available for this study.

### 2.3. Cardiac MRI

All subjects underwent cardiac MRI at our institution using a 3.0 Tesla multi-transmit magnetic resonance imaging system (Achieva, Philips Healthcare, Best, The Netherlands; Release 5.3.1 and 5.6.1) incorporating dStream technology. Vector electrocardiogram-triggered cardiac cine acquisitions were performed on all patients. The maximum gradient performance was 40 mT/m with a slew rate of 200 mT/m/ms. A cardiac phased-array coil was used for signal reception. An axially acquired stack covering the whole heart, a short-axis stack covering the entire left and right ventricles (12–16 slices, no gap) as well as standard two-, three- and four-chamber views were utilized with retrospectively gated cine steady-state free-precession acquisitions (TR/TE/flip angle = 2.7 ms/1.35 ms/42°) for the assessment of heart function in all four cardiac chambers and morphology. A parallel imaging technique with a SENSE-reduction factor of 2 was applied to keep breath-holding times ≤ 12 s. Within one cardiac cycle >25 reconstructed heart frames were acquired in order to achieve greater temporal resolution, as recently demonstrated [22]. The spatial resolution was 1.5 × 1.5 × 8 mm³.

### 2.4. Strain Analysis

Strain analysis was conducted using the CVI42^®^ software package (Circle Cardiovascular Imaging Inc., Calgary, AB, Canada, Release 5.12.1) based on cine steady-state free-precession acquisitions. LA and RA feature-tracking techniques were applied as previously described [23]. The longitudinal axis of the four-chamber view served as the quantitative parameter for LA, RA and RV strain assessment. An additional short-axis quantification was carried out for RV strain and an additional two-chamber view assessment was included for LA strain. The following variables were used to comprehensively assess RV strain: global and regional longitudinal, circumferential and radial strain as well as global peak systolic and diastolic strain rate. RA and LA strain were described by the reservoir, conduit and booster strain and strain rate, respectively. At end-systole and end-diastole, the contours of LA, RA and RV endo- and epicardium were delineated semi-automatically. For RA strain assessment, the linings excluded the superior and inferior ostia of the vena cava as well as the right atrial appendage. For LA strain assessment, the linings excluded the ostia of the pulmonary veins and left atrial appendage. Volumetric LA, RA and RV quantifications were obtained using the disc-summation technique (Simpson approach) at the end of diastole and systole.

### 2.5. Descriptive Statistics

Descriptive statistics were processed in SPSS (version 27.0.0.0, IBM Deutschland GmbH, IBM, Armonk, NY, USA). All continuous variables were presented as mean ± standard deviation (SD) when normally distributed, otherwise as median with interquartile range (IQR). Comparison of baseline characteristics, volumetric parameters and cardiac strain between three different groups was carried out using the univariate ANOVA or ANOVA–Welch test if the criteria were met. A post-hoc Tukey HSD was conducted in case of homogeneity of variance, otherwise a Games–Howell was used to identify inter-group differences. In case of nonparametric data, the Kruskal–Wallis test was used.

### 2.6. Correlation Matrix

For the linear correlation, a matrix was generated. The matrix displays the correlation coefficient for different variables. Numbers in the matrix varied from −1 to +1. A number closer to +1 indicates a strong positive correlation, a number closer to −1 indicates a strong negative correlation and 0 demonstrates no correlation between the features. Initially, the data were normalized for all features. Normalizing the data reduces the bias caused by larger-scale data. The correlation matrix was generated using Pearson’s product–moment correlation.

### 2.7. Classification Algorithms

**Figure 1:**
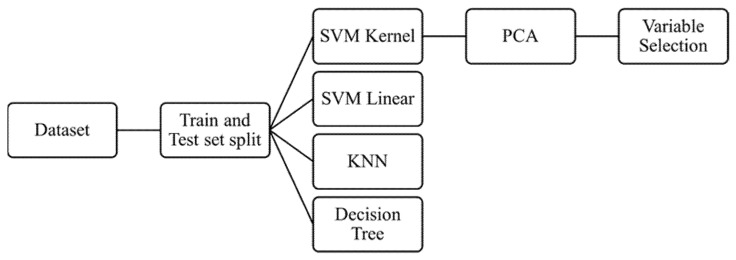
Flow diagram representing the methodology of machine learning and the classifier algorithms used. SVM = supervised machine learning, KNN = k-nearest neighbor, PCA = principal component analysis.

Multiple machine learning classifier algorithms such as k-nearest neighbor (KNN), support vector machine (SVM) linear, SVM kernel RBF, and decision tree (DT) were used on the dataset as recently described in literature [24,25]. The hyperparameters were considered as the default value used by SVC (support vector classifier) which fits the model and returns the “best fit” hyperplane for the data. To estimate the algorithms, training and testing methodology were used and ultimately the performance evaluation was calculated utilizing the accuracy and confusion matrix. A summary of the workflow is presented in Figure 1.

**Figure 2:**
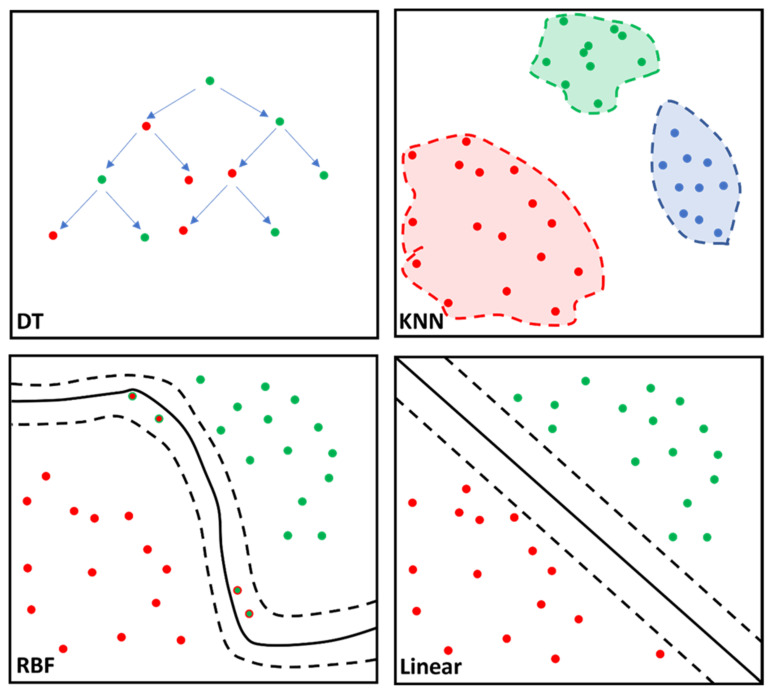
Simplified illustration of the classifier algorithm function for the decision tree (DT), k-nearest neighbor (KNN), support vector machine (SVM), radial basis function (RBF) kernel and SVM linear are portrayed. DT is characterized by the nodes (dots) representing variables and the branches (arrows) representing decisions. KNN calculates data proximity (Euclidian distance) by assuming similar variables remain at a shorter distance from one another. RBF computes similarity between two data points ranging from 0 (similar) to 1 (dissimilar) enabling complex separation between data points. SVM linear separates the data points based on the linear dividing “hyperplane”, albeit with less flexibility than SVM RBF kernel. Different colored dots represent different cohorts; solid line corresponds to best separation of cohorts; dashed lines represent confidence intervals.

Processing of variables for the mentioned algorithms was performed using Python (Version nr. 3.8.12). This study implemented a linear and radial basis function (RBF) SVM, KNN and DT for MRI data analysis. Their basic function is illustrated in Figure 2. The SVM algorithm was used to predict a diagnosis of amyloidotic cardiomyopathy in a cohort with HCM patients and healthy controls. Thus, the SVM classifier predictor variable is cardiac amyloidosis. The data were split into two categories: 80% of the data were used to train and fit the model and the remaining 20% of the data were used to validate the accuracy of the model. Then, the training model was fitted to all the algorithms. Ultimately, the fitted model was validated using the test set.

### 2.8. Support Vector Machine (SVM) RBF

The algorithm, referred to as radial basis function (RBF) kernel, is characterized as a pattern analysis algorithm that utilizes specific vectors referred to as “input spaces” to quantify the degree of similarity between two data points. The RBF kernel value ranges from zero to one. Similarity between points would result in the RBF kernel value being closer to zero, two dissimilar points shift the RBF kernel value closer to 1. Precision (positive predictive value), recall score (sensitivity) and F1-score (test accuracy) were calculated. To calculate the AUC, the one vs. rest (ovr) method was used to evaluate multiclass models by comparing each class against all other classes at the same time.

### 2.9. K-Nearest Neighbors (KNN)

The KNN algorithm [26] was used to measure document relevancy to a given query termed the Euclidean distance. Although numerous techniques exist to conduct this algorithmic analysis, the Euclidean distance remains the most popular due to its efficiency and simplicity. The Euclidean distance is the measure between the query vector and the document vector. This algorithm assumes that similar variables remain in closer proximity. Although this classifier algorithm requires no prior training, its prediction capabilities suffer depending on the data size.

### 2.10. Decision Tree (DT)

A decision tree is another supervised machine learning algorithm that is used in both regression and classification problems. In this algorithm, the data is continuously split based on various parameters, and it can be explained by entities such as decision nodes and leaves. The same training and testing methodology were applied to both KNN and DT.

### 2.11. Principal Component Analysis (PCA)

Principal component analysis is a dimensionality reduction method widely used to reduce the dimensionality of large datasets with a large number of variables improving cost-effectiveness and efficiency while preserving as much ‘variability’ (i.e., statistical information) as possible [27]. Feature selection is based on the magnitude of the correlation coefficients irrespective of their negative or positive sign. The PCA analysis can be based on linear SVM or SVM RBF. Using PCA, the top ten variables with the greatest diagnostic value for CA differentiation (out of the 41 available features in this study) were identified and their cumulative diagnostic performance was assessed.

## 3. Results

### 3.1. Cardiac Features

This retrospective study enrolled a total of 107 subjects, including 43 CA patients, 20 HCM patients and 44 CTRL patients. CA patients made up the oldest cohort (79 years (IQR 71; 85) in contrast to HCM (63.9 ± 7.4 years) and CTRL (56.3 years (IQR 52.5; 62.9)). CA patients showed significantly impaired ejection fraction for both the left and right ventricle compared to CTRL and HCM (all *p* < 0.001). Moreover, CA-associated impairment of cardiac strain was observed in various chambers, for example for the reservoir phase of both atria (RA; HCM: 33.5 ± 16.3% vs. CA: 10.6% (5.6; 19.9), *p* < 0.001; LA (four-chamber); HCM: 14.7 ± 7.1% vs. CA: 7.0% (4.5; 11.1), *p* < 0.001). All features are summarized in Table 1 and Tables S1–S3.

### 3.2. Classification Algorithms

The SVM matrices were generated (Figure S1). Assumed physiological relationships between features could be observed. For instance, the RA reservoir phase (RA_S_Res) and LA reservoir phase (LA_S_Res_2Ch) present a correlation coefficient of 0.68, suggestive of functional bi-atrial interdependency.

**Figure 3:**
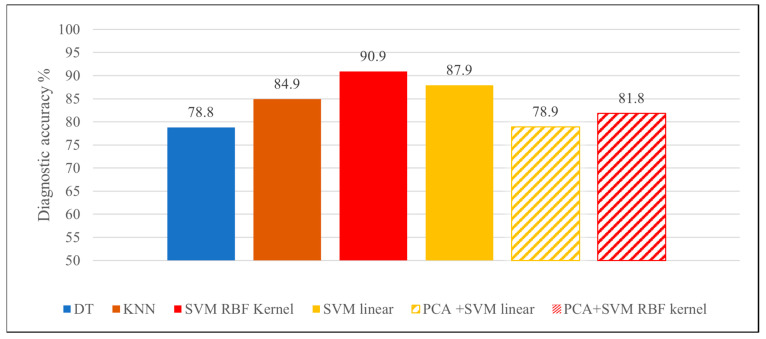
Diagnostic accuracies achieved by the various classifier algorithms.

The accuracies were 84.9% (0.970) for the KNN algorithm, 78.8% (0.787) for the DT algorithm, 87.9% (AUC: 0.962) for SVM linear algorithm and 90.9% (AUC: 0.996) for the SVM RBF kernel (Figure 3).

**Figure 4:**
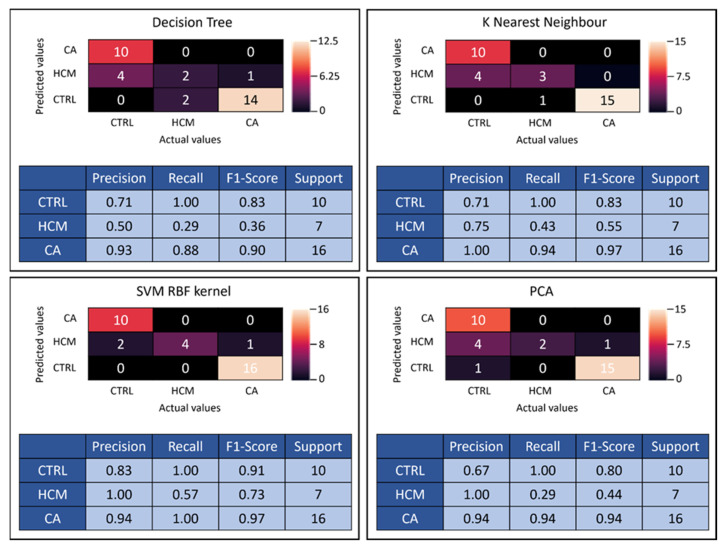
The confusion matrices for 41-feature k-nearest neighbor (KNN), decision tree (DT), SVM radial basis function (RBF) kernel and for 10-feature principal component analysis (PCA + SVM RBF kernel) are presented with corresponding performance statistics. Precision = positive predictive value, recall score = sensitivity, F1-score = test accuracy, Support = number of subjects tested, SVM = support vector machine, RBF = radial basis function, PCA = principal component analysis.

SVM RBF kernel presented the highest accuracy rate among all employed methods, which is why it was the algorithm of choice for PCA. Corresponding confusion matrices and classification reports were generated for all four classifier algorithms (Figure 4). Confusion matrices demonstrate the performance summary of the classifier between the actual and the predicted outcomes utilizing 20% of the data. For example, the confusion matrix for SVM RBF kernel is displayed (Figure 4), whereby out of 17 biopsy-validated CA patients, 1 was predicted false negative, reducing the precision (positive predictive value) to 0.94. For CTRL subjects, 2 were predicted false positive from a total of 12, lowering precision to 0.83. Out of seven HCM patients, three were predicted false negative, resulting in a recall (sensitivity) of 0.57.

### 3.3. Principal Component Analysis (PCA)

**Figure 5:**
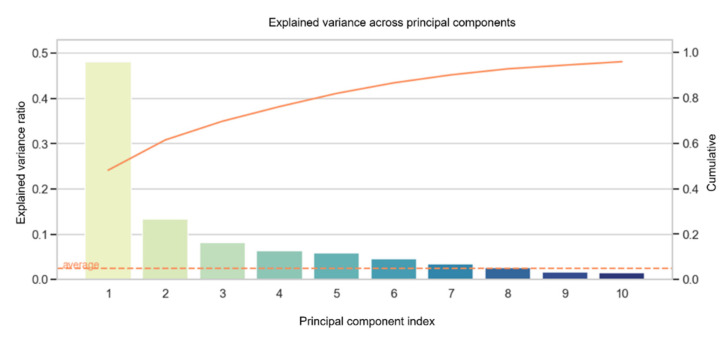
The explained variance of the 10 most valuable diagnostic features is given in descending order from left to right. The PCA bars each represent one component. For example, the first bar of the explained variance is composed of the following variables shown in Table 2. Solid line corresponds to the cumulatively explain of the data. LV-EF = left ventricular ejection fraction, RV-EF = right ventricular ejection fraction, RA = right atrial, LA = left atrial, RV = right ventricular.

The variance across principal components (Figure 5) found that the top ten variables can cumulatively explain 95.9% of the data with an accuracy of 78.9% using the linear SVM and 81.8% using the RBF kernel SVM. The main components are summarized in a matrix (Figure S2). Table 2 represents the top ten highest-rated variables of the first principal component. Among the top ten predictors, LV-EF and RV-EF account for important CA predictors (Table 2 and Figure S2). Moreover, the cardiac strain of the reservoir phase for both the left and right atria appears to contribute high CA predictability (Table 2) corresponding with their significant impairment in wall deformation compared to CTRL and HCM (*p* ≤ 0.003; Tables S1 and S3).

The confusion matrix for PCA using the SVM RBF algorithm is summarized in Figure 4. In contrast to the previous models employing 41 variables, PCA was limited to 10 variables, consequently lowering the recall rate to 0.94 for CA and 0.29 for HCM predictions (Figure 4). Moreover, the precision rate of CTRL was reduced to 0.67. The loss in discriminatory power of using PCA instead of all 41 features is reflected in lowered F1 scores.

## 4. Discussion

To the best of our knowledge, this study is the first to report a supervised machine learning diagnostic approach via CMR left atrial, right atrial and right ventricular quantified strain and general cardiac functions. The novel findings of the present study are as follows: (i) supervised machine learning accurately differentiates CA within a cohort of HCM patients and CTRL subjects, (ii) larger datasets cumulatively contribute towards discriminative performance, (iii) LV-EF and RV-EF remain important feasible clinical predictors of CA due to their routine availability.

### 4.1. Machine Learning vs. State-of-the-Art

A recent left ventricular multiparametric assessment of 41 CA and 45 HCM patients found the diagnostic accuracy-associated AUC to be 82% for percentage normal myocardial strain, 81% for atypical LGE and 94% for the combination of both [6]. A further multi-center-based study observed a diagnostic accuracy-associated AUC of LGE to be as high as 95% [28]. Moreover, the combination of deep learning and LGE–CMR presented good diagnostic accuracy (88%) in CA patients [29]. The power of machine learning elucidated throughout this study detected 90.9% (AUC 0.996) of CA patients based on 41 variables and 82% (AUC 0.962) of CA patients based on the 10 best variables. These findings suggest that the diagnostic accuracy of our machine learning-based approach is able to compete with diagnostic accuracies of the state-of-the-art methods without the incorporation of LGE left ventricular tissue characterization or even left ventricular deformation analysis. LGE and the associated evaluation of the left ventricular myocardium play a pivotal role in the differential diagnostic of the hypertrophic phenotype. However, the administration of gadolinium poses a risk, especially for patients with advanced renal insufficiency. For example, patients with systemic amyloidosis may suffer from renal impairment after progressive amyloid infiltration. Thus, gadolinium administration may be contraindicated, consequently reducing the discriminative power of a CMR examination.

### 4.2. Application of Machine Learning in Cardiac Imaging

The recent integration of machine learning has presented promising gadolinium-free aspects for CA diagnostics. For example, Huang et al. highlighted the potential diagnostic function of texture analysis. A diagnostic accuracy of 82.2% was observed based on seven texture features of the widely available T2-weighted images for CA differentiation from HCM in a similar hypertrophy subgroup using the machine learning algorithm [30]. A similar principle has been applied for native T1 mapping for accurate differentiation between HCM patients and patients with hypertensive heart disease achieving a maximum diagnostic accuracy of 82% [31]. Further non-CA diagnostic developments showed that integrating artificial intelligence in automated chamber quantification for left ventricular strain may detect cardiotoxic remodeling following breast cancer treatment [32]. Moreover, AI-derived strains identified left ventricular global longitudinal strain as a strong predictor of major adverse cardiac events prediction [33]. These observations reiterate the enhanced diagnostic performance of artificial intelligence in non-contrast cardiac wall deformation analysis or myocardial texture assessment. The novelty of our study is underscored by the use of multi-chamber strain diagnostics for CA, without incorporation of the left ventricle or contrast agent, which has not been clinically established. Recent studies have substantiated that amyloidotic infiltration of the myocardium exacerbates cardiac deformation at both the atrial [11,13] and ventricular level [6,15]. This allows for anticipation of a unique cardiac strain profile associated with amyloidotic cardiomyopathy. In the era of increasingly automatized endo- and epicardial contouring, software-based strain evaluation using four-, two-chamber and short-axis cine sequences, multi-chamber volumetrics and strain can be efficiently quantified.

### 4.3. Classifier Algorithms

The choice of classifier algorithm is constantly challenged, as performance is dependent on the complexity and distribution of the dataset. Our results showed that diagnostic accuracy varies depending on the classifier used. In this case, utilizing the SVM linear is simpler and more efficient than RBF, although it results in lowered diagnostic performance as data is not always linearly separable. Although RBF shares advantages with KNN, it often remains superior by overcoming the space complex problem. The power of the RBF kernel lies in its ability to map points in higher-dimensional spaces, which appears best suited for our dataset.

As anticipated, higher diagnostic accuracies are obtained with the 41-variable model; however PCA-associated reduction to a quarter of the variables comes at a cost of only an 8.9% loss in diagnostic accuracy. Thus, depending on the time and effort for parameter quantification the appropriate model can be selected. Moreover, the majority of features in the PCA model were based on left and right atrial strain parameters. Impairment of nearly all functional phases for left and right atrial longitudinal strain appears to be a further valuable discriminative characteristic contributing towards diagnostic accuracy, as previously observed for the left atrium [11]. Additionally, visual characteristics such as atrial enlargement, interatrial septal thickening and atrial dysfunction are generally associated with CA rather than HCM phenotype [14]. These morphological differences appear to be associated with greater atrial strain impairment in CA patients compared to HCM patients. The PCA model highlights their importance in disease discrimination, supported by recent studies [11,13]. Moreover, LV-EF and RV-EF remain strong discriminatory diagnostic features for CA differentiation in agreement with recent studies [15,30]. Ultimately, amyloid infiltration of the myocardium appears to induce greater impairment of sarcomere function than the genetic disorder of HCM for chamber RA, LA and RV.

### 4.4. Clinical Outlook

Integration of machine learning in medical diagnostics will increase in the future as its efficiency for processing large clinical datasets cannot be matched by human cognition. We present an exemplary study in which supervised machine learning of multi-chamber MRI quantified volumetrics and strain data achieved CA prediction for a 41- and 10-variable dataset. Depending on the classifier algorithm employed, diagnostic accuracies ranged from fair to excellent [34]. In this study, the hyperparameters were considered as the default value used by SVC which fits the model and returns the “best fit” hyperplane for the data. Future perspectives may include tuning the hyperparameters via grid search to optimize the choice of algorithms to ultimately raise their diagnostic performance further.

## 5. Limitations

This study is based on a retrospective single-center observational design with typical limitations. The cohorts were not matched, although only healthy subjects above the age of 50 years were included in an attempt to provide greater homogeneity. Moreover, this approach remains an unsupervised algorithmic model. The extent to which this model is applicable in an unsupervised setting is not established. Furthermore, the cohort primarily includes CA patients in advanced stages of the disease and cannot account for early-stage disease detection. Algorithms cannot establish a cause–effect. They can however recognize numerical patterns that emerge from large datasets in order to deliver supportive output to the physician in clinical practice. However, this remains a preliminary study, which requires validation using larger datasets and further risk assessment. Risk assessment can be conducted using a multicriteria decision analysis (MCDA) [35,36].

## 6. Conclusions

Machine learning for a large and a small dataset of RA, LA and RV cardiac strains and functional parameters presents competitive diagnostic accuracies for CA detection when compared to the state-of-the-art methods. The observations of our scientific challenge may introduce new perspectives in non-contrast CMR diagnostics with the potential of clinical decision-making support. The support vector machine algorithm using a radial basis function kernel was found to be the most efficient subheading algorithm achieving competitive diagnostic accuracies under supervised conditions.

## Figures and Tables

**Table 1:** Baseline parameters of patients with cardiac amyloidosis and hypertrophic cardiomyopathy. Comparison to healthy control subjects.

	CTRL	HCM	CA	Post-Hoc Test *p*-Value
N	44	20	43	
Sex [males]	23	10	32	
Age [yrs] ^b^	56.3 (52.5; 62.9) ^a^	63.9 ± 7.4	79 (71; 85) ^a^	CA-HCM, *p* < 0.001 CA-CTRL, *p* < 0.001 HCM-CTRL, *p* = 0.005
BSA [m²] ^c^	1.87 ± 0.20	1.96 ± 0.18	1.89 ± 0.22	n.s.
HR [bpm] ^b^	64.9 ± 10.0	63.1 ± 7.8	74.0 ± 14.8	CA-HCM; *p* = 0.001 CA-CTRL; *p* = 0.004 HCM-CTRL; *p* = 0.706
LV-EF [%] ^b^	66.9 ± 4.7	72.2 ± 8.6	60.0 (50; 65) ^a^	CA-HCM; *p* < 0.001 CA-CTRL; *p* < 0.001 HCM-CTRL; *p* = 0.043

^a^ median value (interquartile range), ^b^ ANOVA–Welch; Games–Howell, ^c^ ANOVA–Tukey–HSDCTRL—healthy control subjects, HCM—hypertrophic cardiomyopathy patients, CA—cardiac amyloidosis patients, BSA—body surface area, HR—heart rate, LV—left ventricular, EF—ejection fraction, n.s.—not significant.

**Table 2:** Top ten highest rates for the first principal component.

Variance Ranking	Magnitude of Coefficients
LA_S_Res_2Ch	0.410296
RA_S_Res	0.381366
LA_S_Res_4Ch	0.276865
RV_EF	0.269950
RV_GRS_4Ch	0.267888
RA_S_Con	0.224486
LA_S_Con_2Ch	0.212670
LA_S_Boo_2Ch	0.197627
LV_EF	0.159073
RA_S_Boo	0.156880

LV_EF = left ventricular ejection fraction, RV_EF = right ventricular ejection fraction, RA_S_Res = right atrial strain of reservoir phase, RA_S_Con = right atrial strain of conduit phase, RA_S_Boo = right atrial strain of booster phase, LA_S_Res_4Ch = left atrial strain of reservoir phase in four-chamber view, LA_S_Res_2Ch = left atrial strain of reservoir phase in two-chamber view, LA_S_Con_2Ch = left atrial strain of conduit phase in two-chamber view, LA_S_Boo_2Ch = left atrial strain of booster phase in two-chamber view, RV_GRS_4Ch = right ventricular global radial strain in four-chamber view.

## Data Availability

The datasets used and/or analyzed during the current study are available from the corresponding author on reasonable request.

## Supplementary Materials

The following supporting information can be downloaded at: https://www.mdpi.com/article/10.3390/diagnostics12112693/s1. Figure S1: The 41-variable matrix with strain measurements from the right atrium, left atrium and right ventricle and with cardiac functional parameters. Figure S2: The 10-variable PCA matrix with the highest weighted diagnostic parameters. Table S1: Right atrial parameters of patients with cardiac amyloidosis and hypertrophic cardiomyopathy. Comparison to healthy control subjects. Table S2: Right ventricular parameters of patients with cardiac amyloidosis and hypertrophic cardiomyopathy. Comparison to healthy control subjects. Table S3: Left atrial parameters of patients with cardiac amyloidosis and hypertrophic cardiomyopathy. Comparison to healthy control subjects.
