# Supplementary material for: A Machine Learning Challenge: Detection of Cardiac Amyloidosis Based on Bi-Atrial and Right Ventricular Strain and Cardiac Function
Source: Diagnostics (Basel). 2022 Nov 4;12(11):2693. doi: 10.3390/diagnostics12112693 (PMC9689404; doi:10.3390/diagnostics12112693)
Supplement: Supplementary file 1 [file diagnostics-12-02693-s001.zip › diagnostics-1977216-supplementary.pdf]

Supplemental material

A machine learning challenge: detection of cardiac amyloidosis based on bi-atrial and right ventricular strain

Jan Eckstein, MD[1], Negin Moghadasi[2], Hermann Körperich, PhD[1], Elena Weise Valdés[1], Vanessa Sciacca, MD[3], Lech Paluszkiewicz, PhD, MD[4], Wolfgang Burchert PhD, MD[1], Misagh Piran, MD[1]

Table S1. Right atrial parameters of patients with cardiac amyloidosis and hypertrophic cardiomyopathy. Comparison to healthy control subjects.

	CTRL	HCM	CA	post-hoc test p-value
**RA-EDV$_i$ [ml/m^2]**[b]	41.5±10.1	40.2±6.8	49.0 (40; 57)[a]	CA-HCM; p=0.002<br>CA-CTRL; p=0.005<br>HCM-CTRL; p=0.043
**RA-ESV$_i$ [ml/m^2]**[d]	21.5 (17; 26)[a]	14.5±3.9	36.0 (25; 39)[a]	CA-HCM; p<0.001<br>CA-CTRL; p=0.001<br>HCM-CTRL; p=0.002
RA-EF [ml/m^2][c]	47.4 (34.0; 55.2)[a]	63.4±9.8	31.3 (20.4; 40.6)[a]	CA-HCM; p<0.001<br>CA-CTRL; p<0.001<br>HCM-CTRL; p<0.001
**RA-S$_{Res}$ [%]**[c]	45.6±15.2	33.5±16.3	10.6 (5.6; 19.9)[a]	CA-HCM; p<0.001<br>CA-CTRL; p<0.001<br>HCM-CTRL; p=0.005
**RA-S$_{Con}$ [%]**[b]	26.3±10.0	19.9±11.7	5.4 (2.8; 9.2)[a]	CA-HCM; p<0.001<br>CA-CTRL; p<0.001<br>HCM-CTRL; p=0.105
**RA-S$_{Boo}$ [%]**[c]	17.1 (13.4; 23.7)[a]	13.6±7.7	5.3±6.5	CA-HCM; p<0.001<br>CA-CTRL; p<0.001<br>HCM-CTRL; p=0.025
**RA-SR$_{Res}$ [s^{-1}]**[c]	2.47±1.01	2.00 (1.10; 2.60)[a]	0.70 (0.40; 1.50)[a]	CA-HCM; p=0.001<br>CA-CTRL; p<0.001<br>HCM-CTRL; p=0.070
**RA-SR$_{Con}$ [s^{-1}]**[b]	-2.01<br>(-3.05; -1.50)[a]	-1.19±0.84	-0.50<br>(-0.70; -0.20)[a]	CA-HCM; p=0.003<br>CA-CTRL; p<0.001<br>HCM-CTRL; p<0.001
**RA-SR$_{Boo}$ [s^{-1}]**[b]	-2.46±0.95	-2.13±0.89	-0.80<br>(-1.40; -0.50)[a]	CA-HCM; p<0.001<br>CA-CTRL; p<0.001<br>HCM-CTRL; p=0.346

[a] – median value (interquartile range), [b] - ANOVA-Welch; Games-Howell, [c] – ANOVA-Tukey-HSD, [d] - Kruskal -Wallis – Test. CTRL – healthy control subjects, HCM – hypertrophic cardiomyopathy patients, CA – cardiac amyloidosis patients, RA – right atrium, EDVi – indexed end-diastolic volume, ESVi – indexed end-systolic volume, S_{Res} – reservoir strain, S_{Con} – conduit strain, S_{Boo} – booster strain, SR_{Res} – reservoir strain rate, SR_{Con} – conduit strain rate, SR_{Boo} – booster strain rate, n.s. – not significant.

Table S2. Right ventricular parameters of patients with cardiac amyloidosis and hypertrophic cardiomyopathy. Comparison to healthy control subjects.

	CTRL	HCM	CA	post-hoc test p-value
**RV-EDV$_i$ [ml/m^2]**[b]	76.0±12.7	70.4±11.9	87.6±20.2	CA-HCM; p<0.001<br>CA-CTRL; p=0.006<br>HCM-CTRL; p=0.214
**RV-ESV$_i$ [ml/m^2]**[b]	30.1±8.1	30.9±10.4	55.4±18.1	CA-HCM; p<0.001<br>CA-CTRL; p<0.001<br>HCM-CTRL; p=0.947
**RV-EF [%]**[b]	60.8±5.8	65.0 (56; 69)[a]	36.4±13.4	CA-HCM; p<0.001<br>CA-CTRL; p<0.001<br>HCM-CTRL; p=0.879
**RV-GRS$_{SAX}$ [%]**[c]	19.0 (15.0;23.6)[a]	16.5 (14.4;21.5)[a]	11.7 (8.5;13.8)[a]	CA-HCM; p<0.001<br>CA-CTRL; p<0.001<br>HCM-CTRL; p=0.610
**RV-GCS$_{SAX}$ [%]**[c]	-11.6±3.1	-9.4 (-13.2;-8.8)[a]	-7.6 (-9.6;-5.6)[a]	CA-HCM; p=0.001<br>CA-CTRL; p<0.001<br>HCM-CTRL; p=0.541
**sys RV-GRSR$_{SAX}$ [s^{-1}]**[b]	0.93 (0.81;1.32)[a]	0.96±0.23	0.63±0.30	CA-HCM; p<0.001<br>CA-CTRL; p<0.001<br>HCM-CTRL; p=0.487
**sys RV-GCSR$_{SAX}$ [s^{-1}]**[c]	-0.67±0.21	-0.63<br>(-0.74;-0.51)[a]	-0.44<br>(-0.64;-0.33)[a]	CA-HCM; p=0.007<br>CA-CTRL; p<0.001<br>HCM-CTRL; p=0.999
**dia RV-GRSR$_{SAX}$ [s^{-1}]**[c]	-0.95<br>(-1.30;-0.81)[a]	-0.78±0.30	-0.54<br>(-0.84;-0.43)[a]	CA-HCM; p=0.283<br>CA-CTRL; p<0.001<br>HCM-CTRL; p=0.010
**dia RV-GCSR$_{SAX}$ [s^{-1}]**[c]	0.62±0.16	0.47±0.12	0.43±0.17	CA-HCM; p=0.676<br>CA-CTRL; p<0.001<br>HCM-CTRL; p=0.002
**RV-GRS$_{4Ch}$ [%]**[b]	47.7 (35.2;58.6)[a]	50.0±24.9	28.8±10.9	CA-HCM; p=0.004<br>CA-CTRL; p<0.001<br>HCM-CTRL; p=0.806
**RV-GLS$_{4Ch}$ [%]**[b]	-19.7±4.7	-21.3±6.7	-16.3<br>(-19.7;-13.0)[a]	CA-HCM; p=0.018<br>CA-CTRL; p=0.003<br>HCM-CTRL; p=0.612
**sys RV-GRSR$_{4Ch}$ [s^{-1}]**[d]	2.68±0.96	2.51 (2.02;3.37)[a]	1.89 (1.43;2.49)[a]	CA-HCM; p=0.060<br>CA-CTRL; p=0.009<br>HCM-CTRL; p=1.000
**sys RV-GLSR$_{4Ch}$ [s^{-1}]**[d]	-1.08<br>(-1.43;-0.94)[a]	-1.20<br>(-1.51;-0.97)[a]	-1.16<br>(-1.57;-0.94)[a]	n.s.
**dia RV-GRSR$_{4Ch}$ [s^{-1}]**[d]	-2.68<br>(-3.44;-2.02)[a]	-2.38±1.07	-1.66<br>(-2.18;-1.44)[a]	CA-HCM; p=0.162<br>CA-CTRL; p<0.001<br>HCM-CTRL; p=0.564
**dia RV-GLSR$_{4Ch}$ [s^{-1}]**[d]	1.24 (0.94;1.49)[a]	1.04 (0.82;1.37)[a]	1.07 (0.88;1.41)[a]	n.s.

[a] – median value (interquartile range), [b] - ANOVA-Welch; Games-Howell, [c] – ANOVA-Tukey-HSD, [d] - Kruskal -Wallis – Test. CTRL – healthy control subjects, HCM – hypertrophic cardiomyopathy patients, CA – cardiac amyloidosis patients, RV – right ventricle, EDVi – indexed end-diastolic volume, ESVi – indexed end-systolic volume, EF – ejection fraction, GRS – global radial strain, GCS – global circumferential strain, GLS – global longitudinal strain, GRSR – global radial strain rate,

GCSR – global circumferential strain rate, GLSR – global longitudinal strain rate, sys – systolic, dia – diastolic, SAX – short axis view, 4Ch – four-chamber view, n.s. – not significant.

Table S3. Left atrial parameters of patients with cardiac amyloidosis and hypertrophic cardiomyopathy. Comparison to healthy control subjects.

	CTRL	HCM	CA	post-hoc test p-value
**LA-EDV$_i$ [ml/m^2]^b**	54.2±8.6	56.8±15.8	52.8±17.4	n.s.
**LA-ESV$_i$ [ml/m^2]^b**	24.5 (20.7;28.7)^a	37.6±11.4	40.6±15.5	CA-HCM; p=0.673<br>CA-CTRL; p<0.001<br>HCM-CTRL; p=0.001
**LA-EF [%]^b**	52.8±5.7	38.3±8.1	27.9 (23.1;36.9)^a	CA-HCM; p=0.028<br>CA-CTRL; p<0.001<br>HCM-CTRL; p<0.001
**LA-S$_{Res_4Ch}$ [%]^b**	31.5 (22.1;43.1)^a	14.7±7.1	7.0 (4.5;11.1)^a	CA-HCM; p=0.001<br>CA-CTRL; p<0.001<br>HCM-CTRL; p<0.001
**LA-S$_{Con_4Ch}$ [%]^b**	16.8 (12.5;26.7)^a	7.9±3.8	5.0 (2.8;7.2)^a	CA-HCM; p=0.010<br>CA-CTRL; p<0.001<br>HCM-CTRL; p<0.001
**LA-S$_{Boo_4Ch}$ [%]^d**	13.5 (8.4;19.1)^a	6.8±4.1	1.9 (0.5;3.5)^a	CA-HCM; p=0.005<br>CA-CTRL; p<0.001<br>HCM-CTRL; p=0.013
**LA-S$_{Res_2Ch}$ [%]^b**	48.3±18.1	18.3±10.5	8.0 (3.9;14.0)^a	CA-HCM; p=0.003<br>CA-CTRL; p<0.001<br>HCM-CTRL; p<0.001
**LA-S$_{Con_2Ch}$ [%]^b**	26.1±11.6	7.7±5.0	4.5 (2.6;8.3)^a	CA-HCM; p=0.202<br>CA-CTRL; p<0.001<br>HCM-CTRL; p<0.001
**LA-S$_{Boo_2Ch}$ [%]^b**	21.7 (14.8;26.9)^a	10.5±6.7	2.3 (0.1;7.1)^a	CA-HCM; p=0.001<br>CA-CTRL; p<0.001<br>HCM-CTRL; p<0.001
**LA-SR$_{Res_4Ch}$ [s^{-1}]^b**	1.5 (1.00;2.40)^a	0.81±0.40	0.40 (0.20;0.60)^a	CA-HCM; p=0.003<br>CA-CTRL; p<0.001<br>HCM-CTRL; p<0.001
**LA-SR$_{Con_4Ch}$ [s^{-1}]^b**	-1.80<br>(-2.30;-1.10)^a	-0.51±0.39	-0.47<br>(-0.70;-0.30)^a	CA-HCM; p=0.869<br>CA-CTRL; p<0.001<br>HCM-CTRL; p<0.001
**LA-SR$_{Boo_4Ch}$ [s^{-1}]^b**	-1.5<br>(-2.30;-0.80)^a	-0.77±0.38	-0.40<br>(-0.60;-0.20)^a	CA-HCM; p=0.005<br>CA-CTRL; p=0.003<br>HCM-CTRL; p=0.027
**LA-SR$_{Res_2Ch}$ [s^{-1}]^b**	2.17±1.05	0.84±0.45	0.60±0.38	CA-HCM; p=0.101<br>CA-CTRL; p<0.001<br>HCM-CTRL; p<0.001
**LA-SR$_{Con_2Ch}$ [s^{-1}]^b**	-2.73±1.33	-0.55±0.35	-0.40<br>(-0.60;-0.20)^a	CA-HCM; p=0.326<br>CA-CTRL; p<0.001<br>HCM-CTRL; p<0.001
**LA-SR$_{Boo_2Ch}$ [s^{-1}]^b**	-2.81±1.45	-1.13±0.74	-0.50<br>(-1.10;-0.20)^a	CA-HCM; p=0.062<br>CA-CTRL; p<0.001<br>HCM-CTRL; p<0.001

^{a} – median value (interquartile range), ^{b} - ANOVA-Welch; Games-Howell, ^{c} – ANOVA-Tukey-HSD, ^{d} - Kruskal -Wallis – Test. CTRL – healthy control subjects, HCM – hypertrophic cardiomyopathy patients, CA – cardiac amyloidosis patients, LA – left atrial, EDVi – indexed end-diastolic volume, ESVi – indexed end-systolic volume, EF – ejection fraction, S_{Res} – reservoir strain, S_{Con} – conduit strain, S_{Boo} – booster strain, SR_{Res} – reservoir strain rate, SR_{Con} – conduit strain rate, SR_{Boo} – booster strain rate, 4Ch – four-chamber view, 2Ch – two-chamber view, n.s. – not significant.

Figure S1. The 41-variable matrix with strain measurements from right atrium, left atrium and right ventricle and with cardiac functional parameters.

	sex	age	BSA	RA_EDVi	RA_ESVi	RV_EDVi	RV_ESVi	HR	LV-EF	RV-EF	RA_S_Res	RA_S_Con	RA_S_Boo	RA_SR_Res	RA_SR_Con	RA_SR_Boo	LA_S_Res_4Ch	LA_S_Con_4Ch	LA_S_Boo_4Ch	LA_S_Res_2Ch	LA_S_Con_2Ch	LA_S_Boo_2Ch	LA_SR_Res_4Ch	LA_SR_Con_4Ch	LA_SR_Boo_4Ch	LA_SR_Res_2Ch	LA_SR_Con_2Ch	LA_SR_Boo_2Ch	RV_GRS_SAX
sex	1.000	-0.186	-0.638	-0.206	-0.214	-0.451	-0.406	0.074	0.351	0.310	0.322	0.281	0.274	0.226	-0.326	-0.202	0.093	0.044	0.168	0.219	0.175	0.238	0.141	-0.047	0.014	0.096	-0.145	-0.244	0.184
age	-0.186	1.000	-0.104	0.330	0.396	0.166	0.530	0.144	-0.403	-0.620	-0.603	-0.523	-0.519	-0.430	0.604	0.474	-0.453	-0.388	-0.442	-0.590	-0.544	-0.559	-0.388	0.358	0.186	-0.544	0.541	0.489	-0.469
BSA	-0.638	-0.104	1.000	0.038	-0.019	0.264	0.164	-0.051	-0.186	-0.126	-0.161	-0.072	-0.229	-0.074	0.177	0.071	-0.066	-0.039	-0.100	-0.189	-0.187	-0.165	-0.148	0.086	0.094	-0.128	0.204	0.220	-0.057
RA_EDVi	-0.206	0.330	0.038	1.000	0.855	0.510	0.420	-0.057	-0.103	-0.217	-0.397	-0.346	-0.339	-0.308	0.345	0.424	-0.185	-0.129	-0.250	-0.242	-0.193	-0.262	-0.198	0.159	0.138	-0.186	0.204	0.282	-0.262
RA_ESVi	-0.214	0.396	-0.019	0.855	1.000	0.545	0.519	0.028	-0.259	-0.393	-0.508	-0.470	-0.397	-0.457	0.395	0.536	-0.262	-0.189	-0.320	-0.301	-0.239	-0.329	-0.230	0.192	0.186	-0.262	0.241	0.310	-0.296
RV_EDVi	-0.451	0.166	0.264	0.510	0.545	1.000	0.769	-0.156	-0.355	-0.409	-0.355	-0.330	-0.275	-0.275	0.303	0.348	-0.109	-0.024	-0.214	-0.193	-0.113	-0.256	-0.170	0.080	0.111	-0.126	0.144	0.212	-0.413
RV_ESVi	-0.406	0.530	0.164	0.420	0.519	0.769	1.000	0.011	-0.582	-0.808	-0.577	-0.473	-0.532	-0.469	0.505	0.518	-0.357	-0.262	-0.429	-0.500	-0.400	-0.543	-0.360	0.269	0.198	-0.417	0.413	0.467	-0.583
HR	0.074	0.144	-0.051	-0.057	0.028	-0.156	0.011	1.000	-0.256	-0.268	-0.197	-0.206	-0.122	-0.135	0.111	0.045	-0.211	-0.189	-0.242	-0.178	-0.146	-0.189	-0.182	0.114	0.063	-0.040	0.039	0.019	-0.175
LV-EF	0.351	-0.403	-0.186	-0.103	-0.259	-0.355	-0.582	-0.256	1.000	0.708	0.438	0.358	0.406	0.453	-0.354	-0.398	0.271	0.220	0.289	0.356	0.272	0.401	0.240	-0.180	-0.123	0.289	-0.267	-0.302	0.509
RV-EF	0.310	-0.620	-0.126	-0.217	-0.393	-0.409	-0.808	-0.268	0.708	1.000	0.613	0.461	0.620	0.529	-0.492	-0.559	0.418	0.316	0.492	0.553	0.443	0.599	0.391	-0.290	-0.199	0.468	-0.438	-0.488	0.634
RA_S_Res	0.322	-0.603	-0.161	-0.397	-0.508	-0.355	-0.577	-0.197	0.438	0.613	1.000	0.900	0.816	0.771	-0.892	-0.807	0.521	0.456	0.524	0.684	0.666	0.608	0.501	-0.397	-0.245	0.599	-0.655	-0.637	0.448
RA_S_Con	0.281	-0.523	-0.072	-0.346	-0.470	-0.330	-0.473	-0.206	0.358	0.461	0.900	1.000	0.482	0.694	-0.848	-0.668	0.455	0.424	0.421	0.628	0.635	0.533	0.433	-0.304	-0.158	0.535	-0.585	-0.558	0.413
RA_S_Boo	0.274	-0.519	-0.229	-0.339	-0.397	-0.275	-0.532	-0.122	0.406	0.620	0.816	0.482	1.000	0.629	-0.667	-0.737	0.445	0.354	0.496	0.542	0.498	0.516	0.432	-0.395	-0.282	0.493	-0.540	-0.540	0.352
RA_SR_Res	0.226	-0.430	-0.074	-0.308	-0.457	-0.275	-0.469	-0.135	0.453	0.529	0.771	0.694	0.629	1.000	-0.695	-0.649	0.376	0.342	0.354	0.461	0.435	0.425	0.362	-0.255	-0.196	0.441	-0.456	-0.450	0.310
RA_SR_Con	-0.326	0.604	0.177	0.345	0.395	0.303	0.505	0.111	-0.354	-0.492	-0.892	-0.848	-0.667	-0.695	1.000	0.691	-0.519	-0.474	-0.477	-0.699	-0.689	-0.613	-0.504	0.429	0.240	-0.633	0.719	0.704	-0.412
RA_SR_Boo	-0.202	0.474	0.071	0.424	0.536	0.348	0.518	0.045	-0.398	-0.559	-0.807	-0.668	-0.737	-0.649	0.691	1.000	-0.398	-0.308	-0.446	-0.543	-0.533	-0.479	-0.396	0.296	0.201	-0.514	0.557	0.539	-0.394
LA_S_Res_4Ch	0.093	-0.453	-0.066	-0.185	-0.262	-0.109	-0.357	-0.211	0.271	0.418	0.521	0.455	0.445	0.376	-0.519	-0.398	1.000	0.945	0.891	0.601	0.591	0.527	0.855	-0.903	-0.797	0.507	-0.603	-0.434	0.373
LA_S_Con_4Ch	0.044	-0.388	-0.039	-0.129	-0.189	-0.024	-0.262	-0.189	0.220	0.316	0.456	0.424	0.354	0.342	-0.474	-0.308	0.945	1.000	0.711	0.540	0.563	0.438	0.797	-0.870	-0.758	0.456	-0.561	-0.396	0.292
LA_S_Boo_4Ch	0.168	-0.442	-0.100	-0.250	-0.320	-0.214	-0.429	-0.242	0.289	0.492	0.524	0.421	0.496	0.354	-0.477	-0.446	0.891	0.711	1.000	0.563	0.495	0.560	0.768	-0.760	-0.705	0.465	-0.522	-0.402	0.415
LA_S_Res_2Ch	0.219	-0.590	-0.189	-0.242	-0.301	-0.193	-0.500	-0.178	0.356	0.553	0.684	0.628	0.542	0.461	-0.699	-0.543	0.601	0.540	0.563	1.000	0.942	0.925	0.561	-0.487	-0.265	0.851	-0.886	-0.826	0.487
LA_S_Con_2Ch	0.175	-0.544	-0.187	-0.193	-0.239	-0.113	-0.400	-0.146	0.272	0.443	0.666	0.635	0.498	0.435	-0.689	-0.533	0.591	0.563	0.495	0.942	1.000	0.744	0.550	-0.494	-0.277	0.825	-0.898	-0.727	0.377
LA_S_Boo_2Ch	0.238	-0.559	-0.165	-0.262	-0.329	-0.256	-0.543	-0.189	0.401	0.599	0.608	0.533	0.516	0.425	-0.613	-0.479	0.527	0.438	0.560	0.925	0.744	1.000	0.494	-0.410	-0.214	0.761	-0.748	-0.822	0.543
LA_SR_Res_4Ch	0.141	-0.388	-0.148	-0.198	-0.230	-0.170	-0.360	-0.182	0.240	0.391	0.501	0.433	0.432	0.362	-0.504	-0.396	0.855	0.797	0.768	0.561	0.550	0.494	1.000	-0.811	-0.659	0.398	-0.543	-0.411	0.417
LA_SR_Con_4Ch	-0.047	0.358	0.086	0.159	0.192	0.080	0.269	0.114	-0.180	-0.290	-0.397	-0.304	-0.395	-0.255	0.429	0.296	-0.903	-0.870	-0.760	-0.487	-0.494	-0.410	-0.811	1.000	0.815	-0.437	0.546	0.359	-0.310
LA_SR_Boo_4Ch	0.014	0.186	0.094	0.138	0.186	0.111	0.198	0.063	-0.123	-0.199	-0.245	-0.158	-0.282	-0.196	0.240	0.201	-0.797	-0.758	-0.705	-0.265	-0.277	-0.214	-0.659	0.815	1.000	-0.254	0.341	0.152	-0.225
LA_SR_Res_2Ch	0.096	-0.544	-0.128	-0.186	-0.262	-0.126	-0.417	-0.040	0.289	0.468	0.599	0.535	0.493	0.441	-0.633	-0.514	0.507	0.456	0.465	0.851	0.825	0.761	0.398	-0.437	-0.254	1.000	-0.880	-0.775	0.424
LA_SR_Con_2Ch	-0.145	0.541	0.204	0.204	0.241	0.144	0.413	0.039	-0.267	-0.438	-0.655	-0.585	-0.540	-0.456	0.719	0.557	-0.603	-0.561	-0.522	-0.886	-0.898	-0.748	-0.543	0.546	0.341	-0.880	1.000	0.807	-0.410
LA_SR_Boo_2Ch	-0.244	0.489	0.220	0.282	0.310	0.212	0.467	0.019	-0.302	-0.488	-0.637	-0.558	-0.540	-0.450	0.704	0.539	-0.434	-0.396	-0.402	-0.826	-0.727	-0.822	-0.411	0.359	0.152	-0.775	0.807	1.000	-0.477
RV_GRS_SAX	0.184	-0.469	-0.057	-0.262	-0.296	-0.413	-0.583	-0.175	0.509	0.634	0.448	0.413	0.352	0.310	-0.412	-0.394	0.373	0.292	0.415	0.487	0.377	0.543	0.417	-0.310	-0.225	0.424	-0.410	-0.477	1.000
RV_GCS_SAX	-0.213	0.404	0.030	0.222	0.253	0.426	0.554	0.103	-0.420	-0.554	-0.385	-0.347	-0.314	-0.273	0.380	0.349	-0.351	-0.263	-0.403	-0.431	-0.314	-0.502	-0.386	0.300	0.214	-0.367	0.365	0.431	-0.900
RV_GRSR_SAX_sys	0.062	-0.499	0.018	-0.220	-0.267	-0.371	-0.571	-0.049	0.481	0.627	0.372	0.326	0.315	0.317	-0.374	-0.411	0.260	0.153	0.335	0.444	0.332	0.507	0.312	-0.241	-0.175	0.461	-0.415	-0.436	0.834
RV_GCSR_SAX_sys	0.002	0.226	-0.026	0.233	0.291	0.359	0.406	-0.033	-0.290	-0.357	-0.255	-0.213	-0.231	-0.269	0.259	0.319	-0.158	-0.084	-0.217	-0.243	-0.164	-0.299	-0.196	0.151	0.099	-0.224	0.254	0.240	-0.539
RV_GRSR_SAX_dia	-0.152	0.446	0.069	0.165	0.178	0.328	0.507	-0.035	-0.381	-0.544	-0.379	-0.333	-0.320	-0.249	0.422	0.333	-0.361	-0.280	-0.394	-0.423	-0.349	-0.448	-0.375	0.360	0.308	-0.449	0.461	0.481	-0.834
RV_GCSR_SAX_dia	0.261	-0.443	-0.138	-0.144	-0.159	-0.374	-0.550	0.176	0.353	0.512	0.381	0.344	0.309	0.256	-0.468	-0.357	0.342	0.268	0.368	0.435	0.369	0.448	0.343	-0.324	-0.280	0.451	-0.465	-0.515	0.700
RV_GRS_4Ch	0.207	-0.298	-0.003	-0.156	-0.277	-0.234	-0.388	-0.139	0.357	0.477	0.506	0.482	0.379	0.444	-0.451	-0.417	0.304	0.246	0.319	0.396	0.346	0.397	0.309	-0.147	-0.061	0.322	-0.330	-0.420	0.514
RV_GLS_4Ch	-0.199	0.138	-0.016	0.055	0.168	0.260	0.332	0.081	-0.244	-0.351	-0.375	-0.374	-0.257	-0.337	0.298	0.310	-0.225	-0.180	-0.251	-0.283	-0.222	-0.312	-0.235	0.113	0.051	-0.208	0.218	0.302	-0.485
RV_GRSR_4Ch_sys	0.165	-0.142	-0.032	-0.081	-0.180	-0.188	-0.217	0.002	0.338	0.271	0.328	0.295	0.268	0.280	-0.304	-0.323	0.168	0.118	0.182	0.193	0.170	0.192	0.108	-0.073	-0.013	0.212	-0.182	-0.247	0.269
RV_GLSR_4Ch_sys	0.023	-0.146	0.084	-0.141	-0.081	0.031	-0.091	-0.254	0.040	0.102	0.025	0.017	0.027	-0.033	-0.038	-0.001	0.124	0.128	0.093	0.045	0.039	0.047	0.087	-0.117	-0.056	0.001	0.008	-0.013	0.127
RV_GRSR_4Ch_dia	-0.253	0.276	0.103	0.125	0.191	0.195	0.288	-0.090	-0.276	-0.305	-0.434	-0.398	-0.344	-0.386	0.488	0.364	-0.286	-0.238	-0.258	-0.374	-0.356	-0.341	-0.267	0.164	0.100	-0.376	0.400	0.468	-0.316
RV_GLSR_4Ch_dia	0.166	-0.004	-0.119	-0.120	-0.186	-0.241	-0.209	0.242	0.119	0.134	0.194	0.191	0.136	0.145	-0.229	-0.188	0.239	0.191	0.243	0.205	0.144	0.244	0.172	-0.180	-0.147	0.253	-0.241	-0.295	0.221
group	0.204	-0.742	-0.052	-0.330	-0.411	-0.303	-0.632	-0.321	0.416	0.699	0.722	0.613	0.639	0.518	-0.705	-0.596	0.662	0.567	0.666	0.798	0.735	0.757	0.594	-0.558	-0.350	0.688	-0.738	-0.674	0.544

	RV_GCS_SAX	RV_GRSR_SAX_sys	RV_GCSR_SAX_sys	RV_GRSR_SAX_dia	RV_GCSR_SAX_dia	RV_GRS_4Ch	RV_GLS_4Ch	RV_GRSR_4Ch_sys	RV_GLSR_4Ch_sys	RV_GRSR_4Ch_dia	RV_GLSR_4Ch_dia	group
sex	-0.213	0.062	0.002	-0.152	0.261	0.207	-0.199	0.165	0.023	-0.253	0.166	0.204
age	0.404	-0.499	0.226	0.446	-0.443	-0.298	0.138	-0.142	-0.146	0.276	-0.004	-0.742
BSA	0.030	0.018	-0.026	0.069	-0.138	-0.003	-0.016	-0.032	0.084	0.103	-0.119	-0.052
RA_EDVi	0.222	-0.220	0.233	0.165	-0.144	-0.156	0.055	-0.081	-0.141	0.125	-0.120	-0.330
RA_ESVi	0.253	-0.267	0.291	0.178	-0.159	-0.277	0.168	-0.180	-0.081	0.191	-0.186	-0.411
RV_EDVi	0.426	-0.371	0.359	0.328	-0.374	-0.234	0.260	-0.188	0.031	0.195	-0.241	-0.303
RV_ESVi	0.554	-0.571	0.406	0.507	-0.550	-0.388	0.332	-0.217	-0.091	0.288	-0.209	-0.632
HR	0.103	-0.049	-0.033	-0.035	0.176	-0.139	0.081	0.002	-0.254	-0.090	0.242	-0.321
LV-EF	-0.420	0.481	-0.290	-0.381	0.353	0.357	-0.244	0.338	0.040	-0.276	0.119	0.416
RV-EF	-0.554	0.627	-0.357	-0.544	0.512	0.477	-0.351	0.271	0.102	-0.305	0.134	0.699
RA_S_Res	-0.385	0.372	-0.255	-0.379	0.381	0.506	-0.375	0.328	0.025	-0.434	0.194	0.722
RA_S_Con	-0.347	0.326	-0.213	-0.333	0.344	0.482	-0.374	0.295	0.017	-0.398	0.191	0.613
RA_S_Boo	-0.314	0.315	-0.231	-0.320	0.309	0.379	-0.257	0.268	0.027	-0.344	0.136	0.639
RA_SR_Res	-0.273	0.317	-0.269	-0.249	0.256	0.444	-0.337	0.280	-0.033	-0.386	0.145	0.518
RA_SR_Con	0.380	-0.374	0.259	0.422	-0.468	-0.451	0.298	-0.304	-0.038	0.488	-0.229	-0.705
RA_SR_Boo	0.349	-0.411	0.319	0.333	-0.357	-0.417	0.310	-0.323	-0.001	0.364	-0.188	-0.596
LA_S_Res_4Ch	-0.351	0.260	-0.158	-0.361	0.342	0.304	-0.225	0.168	0.124	-0.286	0.239	0.662
LA_S_Con_4Ch	-0.263	0.153	-0.084	-0.280	0.268	0.246	-0.180	0.118	0.128	-0.238	0.191	0.567
LA_S_Boo_4Ch	-0.403	0.335	-0.217	-0.394	0.368	0.319	-0.251	0.182	0.093	-0.258	0.243	0.666
LA_S_Res_2Ch	-0.431	0.444	-0.243	-0.423	0.435	0.396	-0.283	0.193	0.045	-0.374	0.205	0.798
LA_S_Con_2Ch	-0.314	0.332	-0.164	-0.349	0.369	0.346	-0.222	0.170	0.039	-0.356	0.144	0.735
LA_S_Boo_2Ch	-0.502	0.507	-0.299	-0.448	0.448	0.397	-0.312	0.192	0.047	-0.341	0.244	0.757
LA_SR_Res_4Ch	-0.386	0.312	-0.196	-0.375	0.343	0.309	-0.235	0.108	0.087	-0.267	0.172	0.594
LA_SR_Con_4Ch	0.300	-0.241	0.151	0.360	-0.324	-0.147	0.113	-0.073	-0.117	0.164	-0.180	-0.558
LA_SR_Boo_4Ch	0.214	-0.175	0.099	0.308	-0.280	-0.061	0.051	-0.013	-0.056	0.100	-0.147	-0.350
LA_SR_Res_2Ch	-0.367	0.461	-0.224	-0.449	0.451	0.322	-0.208	0.212	0.001	-0.376	0.253	0.688
LA_SR_Con_2Ch	0.365	-0.415	0.254	0.461	-0.465	-0.330	0.218	-0.182	0.008	0.400	-0.241	-0.738
LA_SR_Boo_2Ch	0.431	-0.436	0.240	0.481	-0.515	-0.420	0.302	-0.247	-0.013	0.468	-0.295	-0.674
RV_GRS_SAX	-0.900	0.834	-0.539	-0.834	0.700	0.514	-0.485	0.269	0.127	-0.316	0.221	0.544
RV_GCS_SAX	1.000	-0.736	0.498	0.755	-0.700	-0.436	0.545	-0.134	-0.250	0.296	-0.274	-0.498
RV_GRSR_SAX_sys	-0.736	1.000	-0.626	-0.794	0.720	0.405	-0.354	0.191	0.019	-0.247	0.201	0.499
RV_GCSR_SAX_sys	0.498	-0.626	1.000	0.492	-0.453	-0.217	0.135	-0.008	-0.037	0.174	-0.128	-0.309
RV_GRSR_SAX_dia	0.755	-0.794	0.492	1.000	-0.901	-0.394	0.373	-0.257	-0.012	0.362	-0.325	-0.484
RV_GCSR_SAX_dia	-0.700	0.720	-0.453	-0.901	1.000	0.368	-0.369	0.224	-0.013	-0.409	0.404	0.463
RV_GRS_4Ch	-0.436	0.405	-0.217	-0.394	0.368	1.000	-0.819	0.690	-0.085	-0.765	0.480	0.427
RV_GLS_4Ch	0.545	-0.354	0.135	0.373	-0.369	-0.819	1.000	-0.523	0.054	0.542	-0.469	-0.288
RV_GRSR_4Ch_sys	-0.134	0.191	-0.008	-0.257	0.224	0.690	-0.523	1.000	-0.435	-0.669	0.473	0.193
RV_GLSR_4Ch_sys	-0.250	0.019	-0.037	-0.012	-0.013	-0.085	0.054	-0.435	1.000	0.095	-0.107	0.140
RV_GRSR_4Ch_dia	0.296	-0.247	0.174	0.362	-0.409	-0.765	0.542	-0.669	0.095	1.000	-0.630	-0.375
RV_GLSR_4Ch_dia	-0.274	0.201	-0.128	-0.325	0.404	0.480	-0.469	0.473	-0.107	-0.630	1.000	0.151
group	-0.498	0.499	-0.309	-0.484	0.463	0.427	-0.288	0.193	0.140	-0.375	0.151	1.000

RA_EDVI = right atrial end-diastolic volumetric index, RA_ESVI = right atrial end-systolic volumetric index, RV_EDVI = right ventricular end-diastolic volumetric index, RV_ESVI = right ventricular end-systolic volumetric index, HR = heart rate, LV_EF = left ventricular ejection fraction, RV_EF = right ventricular ejection fraction, RA_S_Res = right atrial strain of reservoir phase, RA_S_Con = right atrial strain of conduit phase, RA_S_Boo = right atrial strain of booster phase, LA_S_Res_4Ch = left atrial strain of reservoir phase in 4 chamber view, LA_S_Con_4Ch = left atrial strain of conduit phase in 4 chamber view, LA_S_Boo_4Ch = left atrial strain of booster phase in 4 chamber view, LA_S_Res_2Ch = left atrial strain of reservoir phase in 2 chamber view, LA_S_Con_2Ch = left atrial strain of conduit phase in 2 chamber view, LA_S_Boo_2Ch = left atrial strain of booster phase in 2 chamber view, LA_SR_Res_4Ch = left atrial strain rate of reservoir phase in 4 chamber view, LA_SR_Con_4Ch = left atrial strain rate of conduit phase in 4 chamber view, LA_SR_Boo_4Ch = left atrial strain rate of booster phase in 4 chamber view, LA_SR_Res_2Ch = left atrial strain rate of reservoir phase in 2 chamber view, LA_SR_Con_2Ch = left atrial strain rate of conduit phase in 2 chamber view, LA_SR_Boo_2Ch = left atrial strain rate of booster phase in 2 chamber view, RV_GRS_SAX = right ventricular global radial strain in short axis, RV_GCS_SAX = right ventricular global circumferential strain in short axis, RV_GRSR_SAX_sys = peak systolic right ventricular global radial strain rate in short axis, RV_GCSR_SAX_sys = peak systolic right ventricular global circumferential strain rate in short axis, RV_GRSR_SAX_dia = peak diastolic right ventricular global radial strain rate in short axis, RV_GCSR_SAX_dia = peak diastolic right ventricular global circumferential strain rate in short axis, RV_GRS_4Ch = right ventricular global radial strain in 4 chamber view, RV_GCS_4Ch = right ventricular global circumferential strain in 4 chamber view, RV_GRSR_4Ch_sys = peak systolic right ventricular global radial strain rate in 4 chamber view, RV_GCSR_4Ch_sys = peak systolic right ventricular global circumferential strain rate in 4 chamber view, RV_GRSR_4Ch_dia = peak diastolic right ventricular global radial strain rate in 4 chamber view, RV_GCSR_4Ch_dia = peak diastolic right ventricular global circumferential strain rate in 4 chamber view

Figure S2. The 10-variable PCA matrix with the highest weighted diagnostic parameters.

	LV_EF	RV_EF	RA_S_Res	RA_S_Con	RA_S_Boo	LA_S_Res_4Ch	LA_S_Res_2Ch	LA_S_Con_2Ch	LA_S_Boo_2Ch	RV_GRS_4Ch	group
LV_EF	1	0,708306509	0,438373749	0,35810266	0,405889033	0,27066212	0,356144169	0,271513214	0,401484108	0,357414287	0,41618219
RV_EF	0,708306509	1	0,613352664	0,461492263	0,620229839	0,417919234	0,553016887	0,443046297	0,599276264	0,476815677	0,699379739
RA_S_Res	0,438373749	0,613352664	1	0,899693475	0,816335614	0,521496659	0,684251587	0,66636702	0,608422154	0,506476621	0,722375067
RA_S_Con	0,35810266	0,461492263	0,899693475	1	0,48232626	0,454669778	0,628053873	0,634587478	0,532612831	0,481961748	0,612703779
RA_S_Boo	0,405889033	0,620229839	0,816335614	0,48232626	1	0,444924727	0,542122864	0,497588138	0,516233416	0,37867228	0,638937464
LA_S_Res_4Ch	0,27066212	0,417919234	0,521496659	0,454669778	0,444924727	1	0,60056813	0,59107024	0,527032432	0,303999213	0,662114635
LA_S_Res_2Ch	0,356144169	0,553016887	0,684251587	0,628053873	0,542122864	0,60056813	1	0,941668607	0,925427434	0,395658199	0,798075756
LA_S_Con_2Ch	0,271513214	0,443046297	0,66636702	0,634587478	0,497588138	0,59107024	0,941668607	1	0,743921986	0,345541495	0,734889557
LA_S_Boo_2Ch	0,401484108	0,599276264	0,608422154	0,532612831	0,516233416	0,527032432	0,925427434	0,743921986	1	0,396595393	0,757289293
RV_GRS_4Ch	0,357414287	0,476815677	0,506476621	0,481961748	0,37867228	0,303999213	0,395658199	0,345541495	0,396595393	1	0,426986905
group	0,41618219	0,699379739	0,722375067	0,612703779	0,638937464	0,662114635	0,798075756	0,734889557	0,757289293	0,426986905	1

LV_EF = left ventricular ejection fraction, RV_EF = right ventricular ejection fraction, RA_S_Res = right atrial strain of reservoir phase, RA_S_Con = right atrial strain of conduit phase, RA_S_Boo = right atrial strain of booster phase, LA_S_Res_4Ch = left atrial strain of reservoir phase in 4 chamber view, LA_S_Res_2Ch = left atrial strain of reservoir phase in 2 chamber view, LA_S_Con_2Ch = left atrial strain of conduit phase in 2 chamber view, LA_S_Boo_2Ch = left atrial strain of booster phase in 2 chamber view, RV_GRS_4Ch = right ventricular global radial strain in 4 chamber view
